# Wheel of Life: self-care and continuing education tool for mental healthcare professionals

**DOI:** 10.1590/0034-7167-2025-0149

**Published:** 2025-11-20

**Authors:** Johnatan Martins Sousa, Joyce Soares Silva Landim, Marciana Gonçalves Farinha, Roselma Lucchese, Camila Cardoso Caixeta, Thatianny Tanferri de Brito Paranaguá, Fernanda Costa Nunes, Ana Lúcia Queiroz Bezerra

**Affiliations:** I Universidade Federal de Goiás. Goiânia, Goiás, Brazil; II Universidade Federal de Uberlândia. Uberlândia, Minas Gerais, Brazil; III Universidade Federal de Catalão. Catalão, Goiás, Brazil; IV Universidade de Brasília. Brasília, Distrito Federal, Brazil

**Keywords:** Self Care, Life, Patient-Centered Care, Continuing Education, Community Mental Health Services., Autocuidado, Vida, Atención Centrada en el Paciente, Educación Continua, Servicios Comunitarios de Salud Mental.

## Abstract

**Objectives:**

to analyze the use of the Wheel of Life as a self-care and Continuing Health Education tool for mental healthcare professionals in psychosocial care settings.

**Methods:**

qualitative intervention research guided by the Experiential Learning Cycle. Four workshops were held with 30 professionals from two Psychosocial Care Centers in central Brazil. Data collection involved the use of a professional profile questionnaire, the Wheel of Life, and field diary notes. The data were subjected to thematic content analysis.

**Results:**

two categories emerged: Repercussions of the Wheel of Life for self-care; and Wheel of Life as an instrument for managing problems.

**Final Considerations:**

the study showed that the Wheel of Life is a tool that can be combined with Continuing Health Education processes, in addition to promoting reflections among professionals on the importance of practicing self-care to care for others.

## RESUMEN

**Objetivos:**

analizar el uso de la Rueda de la Vida como herramienta de autocuidado y educación continua para la salud para profesionales de la salud mental en el ámbito de la atención psicosocial.

**Métodos:**

investigación de intervención cualitativa guiada por el Ciclo de Aprendizaje Experiencial. Se realizaron cuatro talleres con 30 profesionales de dos Centros de Atención Psicosocial del centro de Brasil. La recopilación de datos se realizó mediante un cuestionario de perfil profesional, la Rueda de la Vida y anotaciones en diarios de campo. Los datos se sometieron a un análisis de contenido temático.

**Resultados:**

surgieron dos categorías: Impacto de la Rueda de la Vida en el autocuidado; y La Rueda de la Vida como herramienta para la gestión de problemas.

**Consideraciones Finales:**

el estudio demostró que la Rueda de la Vida es una herramienta que puede combinarse con procesos continuos de educación para la salud, además de promover la reflexión entre los profesionales sobre la importancia de practicar el autocuidado para cuidar a los demás.

## INTRODUCTION

The constant pursuit of knowledge is innate to every human being in society, seeking answers to various questions. However, complacency based on common sense and deep-rooted prejudices can hinder the development of new knowledge, as people begin to believe in unquestionable “truths”^(1)^.

To transform this scenario of stagnation in the work context, Continuing Health Education (CHE) is a resource that generates changes in the training of various professionals, combining training institutions, healthcare service management and teams to improve work processes, as scientific evidence points to the importance of CHE actions in community mental healthcare services for the improvement of practices and implementation of person-centered care^(2)^.

In this regard, researchers highlight the importance of transformations and innovations in teaching methodologies for a critical, reflective practice that respects the uniqueness of others, including mental health training^(3)^. One strategy that can be used in CHE processes is the Wheel of Life (WL), as demonstrated by a study that used this tool to promote leadership development in Primary Health Care (PHC) nurses^(4)^.

WL is a graphical tool in pie format with a scale of 1 to 10 in which a person must qualitatively assess and assign a score to each area of their life in a biopsychosocial way to support interventions for the aspects analyzed^(5)^.

In addition to being a mediating instrument in CHE processes, WL can be considered a self-care tool because it allows a person performing it to identify the strengths and weaknesses of all areas of their life in the present moment. Research described the impact of a Reiki intervention on nursing professionals’ well-being and self-reported health, who used WL as a data collection tool, demonstrating that after the intervention, participants’ emotional well-being improved^(6)^.

In psychosocial care settings, self-care actions for professionals are essential to promote teams’ physical and mental health. A cross-sectional, exploratory, and descriptive observational study describing the lifestyle and mental health of 47 workers at Psychosocial Care Centers (In Portuguese, *Centros de Atenção Psicossocial* - CAPS) I and II in the northern region of the state of Tocantins, Brazil, revealed that although most were classified as having a good quality of life, common mental disorders were identified in the group. The prevalence found was also higher than in other studies of healthcare professionals^(7)^.

Considering the above, research on the construction of knowledge about strategies, methods and actions involving CHE planning and implementation is still scarce^(8)^. Considering that it is not enough to simply promote training processes within mental health teams, it is important to combine these moments of knowledge construction with self-care practices, especially in the context of mental health, as scientific evidence indicates that mental healthcare professionals are susceptible to illness related to work settings^(9)^. Therefore, we aimed to answer the following guiding question: what are the repercussions of using WL for professionals’ self-care and CHE at CAPS?

## OBJECTIVES

To analyze the use of WL as a self-care and CHE tool for mental healthcare professionals in psychosocial care settings.

## METHODS

### Ethical aspects

The research is part of an anchor project approved by the Research Ethics Committee of the *Hospital das Clínicas, Universidade Federal de Goiás*. The guidelines of Resolution 466 of 2012 of the Brazilian National Health Council were followed and, to preserve participants’ anonymity, all were coded by the letter P and order of speech in the group meetings (P1 to P30).

### Study design

This is intervention research with a qualitative approach, which is indicated when the aim is to understand the meanings, experiences and experiences of different actors within institutions, associating a psychosociological intervention during the study to encourage institutional transformation^(10)^. The study was guided by the Experiential Learning Cycle (ELC)^(11)^ and the Person-Centered Clinical Method (PCCM) theoretical framework^(12)^. The study report followed description COnsolidated criteria for REporting Qualitative research recommendations^(13)^.

### Study setting

The research was carried out at two CAPS in the central region of Brazil, one being a Psychosocial Care Center for Alcohol and other Drugs (In Portuguese, *Centro de Atenção Psicossocial álcool e outras drogas* - CAPSad) type III and one Psychosocial Care Center for Children and Adolescents (In Portuguese, *Centro de Atenção Psicossocial infantojuvenil* - CAPSi).

### Data source

Thirty professionals participated, 15 from each CAPS, selected by convenience, based on the inclusion criterion of providing care to patients and their families. Employees who were officially away from work due to vacation or leave, or who worked exclusively in administrative activities, were excluded.

### Data collection and organization

To construct the data, four group meetings were implemented from October to December 2022 in a workshop format that followed the ELC stages: activity (implementation of an experience); analysis (diagnosis of the situation that was experienced through the feedback left by the group); conceptualization (exposition of theory on the topic addressed); and connection (establishment of relationships between everything that was experienced and personal and professional life)^(11)^. All meetings lasted three hours, took place 15 days apart, and were held in the CAPSad III meeting room, where CAPSi professionals traveled.

The data for this study come from the third meeting of the training process, which aimed to work with professionals on the second and third components of PCCM: “Understanding the whole person”; and “Finding common ground”^(12)^.

At the beginning of the meeting, group members were provided with snacks, stationery for creating a personal name tag, and a professional profile questionnaire and Informed Consent Form were provided. Immediately afterward, as a welcoming and warm-up strategy, the group was suggested the “My Superpower” technique, in which the question was asked: Thinking about your role as a mental healthcare professional, what superpower would you like to have? Each professional was given a slip of paper to record their superpower, and then each explained why they chose their power.

During the ELC activity stage, the WL^(14)^ technique was proposed to professionals to work on the second component of PCCM. This technique aimed to analyze the dimensions of group members’ lives, including spirituality, health and disposition, intellectual development, emotional balance, career and work, finances, social contribution, friendships and family, affection and love, social life, fun and recreation, and personal development and happiness. Each professional received a sheet with WL, markers, and colored pencils to color their wheel in ten minutes.

After completing WL, the group was asked if anyone would like to share their work with others, guided by the following questions: what was it like experiencing the activity? How does what you experienced relate to your personal life and work?

Subsequently, to work on the third component of PCCM, the group was invited to collectively develop a Singular Therapeutic Project (In Portuguese, *Projeto Terapêutico Singular* - PTS) for one of the participants, using WL as a guiding principle. A professional volunteered. After systematizing the PTS to problematize the topic, the following questions were asked: what was it like experiencing the activity? What were the challenges and advantages? How do you collectively construct care for the user? What strategies? Tools? Are users and family members included in this process? Who develops this care plan?

The meetings were mediated by two professionals: a nurse with a master’s degree, specializing in mental health, psychiatric nursing, and group dynamics and team management; and a psychologist with a PhD in health sciences and specializing in consulting and group management.

### Data analysis

The entire process was recorded in audio format and in field diary entries and was subjected to thematic content analysis according to the stages recommended by Bardin^(15)^: pre-analysis, marked by the organization and selection of materials to be analyzed, as well as by data skim reading to support the initial hypotheses; material exploration, which consists of coding the data through the identification of recording and context units, which are grouped by similarity to construct the cores of meaning; and treatment of results: inference and interpretation, which is the dissemination of information analyzed through categories 1 (Impacts of the Wheel of Life for self-care) and 2 (Wheel of Life as an instrument for managing problems).

## RESULTS

### Sociodemographic characterization

Twenty-eight of the 30 professionals who participated in the study were female (93%). The largest age group was 30 to 49 years old (19 participants; 63%), and professional backgrounds were diverse (psychologist (11), nursing technician (8), nurse (3), pharmacist (2), physiotherapist (2), social worker (1), pedagogue (1), music therapist (1), physical education professional (1)).

### Content analysis

As a result of the content analysis process, two categories were constructed (1. Repercussions of the Wheel of Life for self-care; 2. Wheel of Life as an instrument for managing problems), which demonstrate the consequences of using WL with mental health teams.

### Category 1. Repercussions of the Wheel of Life for self-care

Participants expressed that through WL they were able to realize the importance of recognizing themselves as human beings, and that they also have weaknesses like the people they serve:

*When I worked at the BHU, I had a fever and I went to do triage, and a patient looked at me and said, “Wow, doctor also gets sick?”, and then I told her, “First, I’m not a doctor, second, I’m flesh and blood just like you”.* (P2)

*And people, it’s great to become human, to meet another human, which is this person-centered care thing, and it’s really cool.* (P6)

WL allowed the group to understand that, even in their role as caregivers, professionals are also susceptible to illness and that accepting such personal weaknesses humanizes the care provided:

*Before, my insecurity as a new professional was like, “Wow, I can’t get sick. I have to be strong and all that”. But with life experience, we see that no, the more human and sicker you are, the more you bond, you create a connection with your patient* [...]. (P6)

Among the professionals, one participant explained that he uses WL resource in his services, and that its effectiveness is enhanced when he shares his own experiences with the adolescents so that they feel confident in revealing their issues:

*And it’s a good technique, both individually and in groups. I work with teenagers, and we try to extract information from them, but if we only try to extract information without giving anything in return, they shut down. So, what do I do? I talk to them, tell them my life story, what happened to me, and after that, they start to give back.* (P12)

WL proved to be relevant because it allowed for a situational diagnosis of the current moment in professionals’ lives by identifying the most satisfactory areas and those that needed greater attention from the participants:

[...] *lame, because, really, there are some areas that I put here* [...] *emotional balance, I put it at maximum, but then, when I looked at my health and disposition, I realized that health and disposition are part of this emotional balance. Then I saw a discrepancy, I said, “Whoa!” It made me reflect that for me to be here in this ten of emotional balance. I have to go back to my health and take better care of it, because this also contributes to emotional balance* [...]. (P8)

WL helped a participant visualize possible causes that were preventing his wheel from turning, such as fears from the past that were hindering his love life:

*And the issue of affection, which is what Selma also mentioned, how long has it been since I opened myself up to a relationship for fear of what happened before happening again? So, those things, right? But, like, it’s good to look at the things that can change* [...]. (P2)

Participants’ testimonies revealed that WL made them aware of issues such as self-knowledge, the need to look at themselves, and take their own perspective into consideration, not what society dictates:

*I tried to focus more on myself, like, not looking so much* [...] *because, if I were to look at what society itself demands, I think I would be way below.* (P11)

*I find this experience of us doing the Wheel of Life very interesting, because for me, it was a moment when I was more silent, because it was a more reflective thing. Because I have been observing my life, but when you look at it on paper, you are there reflecting on it, you are getting an idea of what you don’t know, so you can really see what areas you are catching* [...]. (P7)

One of the professionals brought up the reflection that it is necessary to be well with oneself in order to be able to take care of users:

[...] *I became more reflective because of this, because of these things you see that affect your emotions. You say, “Wow, it’s bad here”. That’s what you said, when we express our emotions, what we’re experiencing, and you see that it improves in you, it reflects on the person you’re helping* [...]. (P7)

WL highlighted the need to practice empathy during mental healthcare, as everyone experiences difficulties throughout their life journey:

*It was really good, because we deal with a lot of problems with users being admitted and released on the same day* [...] *and, for us professionals, this is frustrating. They’ll go back to the streets, they’ll go back to causing problems for their families and for society. So, the conversation was good, because we realize that being human, everyone has their own moment, has their own variables, so it was very useful for me and my colleagues, because we talked among ourselves.* (P23)

Another important contribution of WL was the recognition by one of the participants of what was already being done to improve what was not so good in his life:

*In my case, I need health and energy. I’m already moving, I’m already in the process with a nutritionist. So, that’s my point: health. I’ve already started an intervention. In my case, if I make a change here, it will affect others. If I make a change here, it will improve.* (P8)

### Category 2. Wheel of Life as an instrument for managing problems

**Figure 1:**
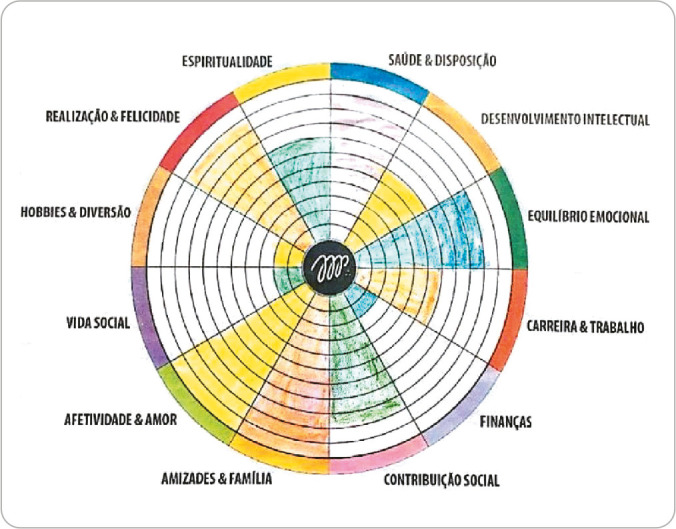
Wheel of Life of a professional member of the group, Aparecida de Goiânia, Goiás, Brazil, 2022

*Source: Pavlo^(14)^.*

After all group members completed their WL, one participant allowed her work to be used to collectively construct a PTS to practically explain the third component of PCCM: “Finding common ground”. Figure 1 presents the professional’s WL.

Through WL, the professional was able to identify which areas of her life were most critical, such as finances, career and work, social life, and fun and recreation:

*My finances aren’t good, I have no social life, no fun, no money, my finances are in shambles.* (P3)

*The social and fun aspects are even more messed up.* (P3)

*I work, work, work. Career and work are higher.* (P3)

When showing the group that gave a very high score to the field of emotional balance, the feedback from colleagues highlighted contradictions between what was said and what was real:

*And how is your balance high?* (P12)

*My husband said I’m very aggressive. I said, “Man, don’t say I’m aggressive”.* (P3)

Another area of life that presented contradictions between the high score and how they actually present themselves was the issue of happiness and personal development, in which a coworker who works with the professional pointed out inconsistencies:

*I hadn’t even seen this here, her life like this and her personal development and happiness is complete.* (P6)

WL helped professionals visualize which areas of their lives needed greater investment, such as leisure and fun, and also enabled them to reflect on possible solutions, such as balancing career and motherhood to improve their finances:

*It needs to improve, but to improve, you have to improve other things first. Finances, career, and work take off.* (P3)

*Right now, I need to balance motherhood. I have an eight-month-old baby and I need to work, but I have to balance it with my husband, with his time, because she spends time with me and with him* [...] *we take turns.* (P3)

Making concessions in her life project over other people’s desires, such as her husband, was also signaled by the professional when problematizing her WL:

[...] *but now he* [my husband] *doesn’t want to work for himself anymore; he wants to work under the CLT. And so, for him to work under the CLT, I’ll have to give up one of my jobs. I have two jobs.* (P3)

Another paradoxical situation highlighted in WL was significant scores regarding affection and love. However, the professional expressed the lack of private moments with her husband and that most of her time is spent on work and caring for her daughter:

*Full, here it is full. Affection, love, friendship, family.* (P3)

*In terms of affection, in terms of love, I think I looked more at the issue of my family, my daughter, my friends, and one kind of responded to the other, right?* (P3)

Regarding friendships, the professional expressed that to avoid weakening the bond, she invites her coworkers to visit her home or make video calls:

*I socialize with friends at work, via video call, when someone comes over to my house.* (P3)

Facilitators asked the professional if WL were the wheel of time, which area would have the highest score, and she answered “work”. Later, she was asked if it were the wheel, what would be most important, and she answered “family”. Given these answers, they asked if one wheel had to overlap the other, if that would be possible, and the professional shook her head:

*You have to find a way to reconcile.* (P3)

After the professional presented WL, the facilitators provided two sheets with copies of the PTS structure, which was also projected onto the room wall using a projector to help the entire group formulate care strategies:

*As a close friend, when we suggest something, she says, “No, come over to my house”. It’s easier to have her over than to go out. We wanted her to go to the park with the baby, but we see here, I think it’s not even due to a lack of conditions, but because she’s just sad and tired.* (P2)

*So, I noted down possible problem situations: physical fatigue, little sleep, sleep deprivation, unrefreshing sleep, difficulty expressing herself, asking for help, seeking help, dissatisfaction, sadness, exhaustion, difficulty, isolation, lack of social interaction, financial hardship, reduced or no support network, and little time for herself.* (P6)

To alleviate the burden, the group suggested possible actions to minimize losses, such as making it possible for their daughter to enroll in a Municipal Early Childhood Education Center (In Portuguese, *Centro Municipal de Educação Infantil* - CMEI):

*And then, when we put this in, I’ll talk about this rest, and then, how is it possible, in the current configuration, to have rest, if we think about the lack of a support network? That there is no family? So, how can there be a support network? So, I have to add CMEI, which will be the time to care for this baby and that will give her and her husband time so that she will feel less guilty and will be able to organize herself from there, to the detriment of other things.* (P6)

Another possibility raised was that friends would take turns caring for the baby. However, the professional pointed out that because of the jealous behavior, the father does not authorize it:

*And then there’s this whole issue of the father needing to understand what this jealousy is, this insecurity about leaving his daughter with strangers. He could see who the people in his circle of friends are that he would trust, so he could share things with them. And then there’s another demand, which is to think about strategies beyond the CMEI.* (P6)

Mental healthcare was suggested for the professional as she is resistant to asking for help, which can trigger emotional distress:

*Another thing would be to urgently refer her to the Mental Health Center. She needs therapy because she has this tendency to shut down, to not be able to ask for help, to want to handle everything alone. So, emotionally, it would be a relief if she allowed herself to not be able to handle it, to not be so hard on herself, or to understand this movement a little better, to be more compassionate with herself, to open up the space.* (P6)

Even with all the efforts to build an assertive PTS for the colleague, the importance of considering the professional’s desire to adhere to what was formulated emerged in the group, which aligns with person-centered care:

*We’re talking about timing because the baby will be at CMEI. And then, we’ll outline the priority levels. If you want, you can think about that too, because we think like this: “There are things that are ideal, tangible, available, now you’ll decide whether or not you want these possibilities”.* (P6)

## DISCUSSION

The “Repercussions of the Wheel of Life for self-care” category showed that the use of this tool with health professionals was to make them see themselves as human beings, equally to CAPS users. A person’s self-concept, through their perceptions, beliefs, values, skills, and more, influences how each individual behaves in their environment^(16)^. Therefore, by recognizing yourself as a human being in front of the people you serve, mental healthcare becomes more humanized.

WL also led mental healthcare professionals to recognize that they are also susceptible to illness, just like the people they serve. An integrative literature review describing the health-illness process in mental healthcare professionals revealed that working conditions can affect their quality of life and that no recent studies addressing this topic were found in the scientific literature^(17)^.

It was shared that WL is also used by one of the professionals with adolescents, and when they express their concerns, users feel more comfortable talking about their lives. In a therapeutic process, when a person is unconditionally accepted as they are, treated empathetically, and feels in a safe environment, they move towards developing personal autonomy and become self-responsible, becoming free^(18)^. Therefore, when using WL, professionals also look at themselves and reflect on their lives, which strengthens the bond with the user.

WL allowed participants to identify areas of their lives that were improving compared to others that needed greater investment as well as to visualize possible causes of what was not so good at that current moment. The movement of focusing on the present moment is a seldom-practiced activity. One possibility that favors focusing on the present and that can be used by professionals is WL, as demonstrated by a study conducted with medical students who used this resource to identify the strengths and weaknesses of a patient’s current life moment in the PHC context^(19)^.

WL awakened professionals to reflections on self-awareness, turning inward, and looking at their own perspective rather than what society preaches. In the healthcare context, self-awareness is one of the desirable attributes for implementing person-centered care, as a professional treating a person with an illness can only understand that person if they first know themselves^(12)^.

The intervention helped the group realize that in order to care for others, it is first necessary to care for oneself. Healthcare professionals are also subject to psychological distress and need to undergo care^(20)^. Therefore, self-care is essential for maintaining both the physical and mental health of teams who routinely deal with others’ suffering.

Another impact of WL on the group was the awareness of practicing empathy during their work practice at CAPS. A key aspect of practicing person-centered care is the use of empathy by those providing care to individuals, families, and communities^(21)^. Therefore, it is necessary for multidisciplinary teams working at CAPS to invest in these relational care technologies for better mental healthcare.

In addition to providing a situational diagnosis of professionals’ lives, WL also enabled them to visualize what they were already doing to change this reality. WL is an assessment tool that allows for the analysis of key areas of life, which are sliced and visually represented in graphic form. This represents individuals’ perspective on their current life and how they would like it to be^(22)^.

The WL category as a tool for problem management demonstrated, through the explanation of WL by one of the professionals, that this resource can be used for this purpose at CAPS. The technique enabled patients to identify the critical areas of their life that consequently affect their mental health.

For a person to truly have mental health, it is not enough to simply be free from mental disorders or psychological distress. Mental health involves a balance between internal and external factors, including family, work, social life, romantic relationships, and coping with frustrations. Therefore, a balance between all these aspects confers mental health^(19)^.

WL made professionals stop and reflect on her own life, which often, due to the countless demands and responsibilities of daily life, does not leave time for self-care. In today’s world, due to the rush of everyday life and people’s unbridled search for something outside of themselves, individuals end up in a state of suffering, as they forget themselves and their emotions, which leads to self-abnegation and triggers robotic behavior^(23)^. Therefore, these are important moments of reflection on one’s own life to recover what is truly important to a person and break out of automatism.

WL allowed professionals to identify which area of their lives had the highest work-related score, compared to others. Work-related stress creates imbalance in people’s lives, as excessive demands, nervousness, extreme exhaustion, and irritability can lead to illness with mood swings, decreased productivity, and impaired interpersonal relationships and quality of life, all of which hinder the full expression of happiness^(23)^.

This finding demonstrates a denial of a person that ultimately leads to suffering. Therefore, when practicing person-centered care, it is important that professionals value and respect the preferences of those they serve, not the convenience of the team^(21)^. By ignoring the real wishes of users, workers will be reinforcing this movement of passivity in people’s lives.

Professionals’ WL also highlighted the difficulty of balancing motherhood with other areas of their lives, such as work and personal life. Since the beginning of time, women have always assumed the role of caregivers, and when placed in this position, they often do not question it, which has become ingrained in society as a moral value related to the female universe. Even with some recent advances, childcare and household chores are still largely the responsibility of women^(24)^. When women work outside the home, it creates a double shift that takes up time that they can dedicate to other areas of their lives, such as their romantic relationships.

Regarding friendships, the professional stated that, to avoid damaging her bonds with friends, she invites them to her home. Friendships are important for people’s psycho-emotional development and can even influence the development of mental disorders. An integrative review that analyzed scientific literature on friendships in people with eating disorders revealed that good-quality bonds reduced the frequency of symptoms, while friendships that made negative comments about a person’s body and eating habits favored the development of disorders^(25)^. Therefore, it is important to consider that, like other areas of life, the field of friendships also needs investment.

After WL presentation, the group created the coworker’s PTS, considering interventions to minimize or solve the problems that emerged. WL is a tool that can mediate the PTS development, as it allows a person seeking help to reflect on what is best for them, according to their own perspective^(19)^. Therefore, this movement of turning to oneself contributes to the realization of care centered on a person, not on the desires and wishes of a healthcare professional.

At CAPS, a powerful strategy for formulating action plans to meet users’ needs is the PTS. When developed collectively, the PTS strengthens the bonds established between the team, users, and their families, as user involvement in the process allows for the development of their autonomy during healthcare^(26)^. Therefore, it is important to give users a voice, not only to identify their problems, but also to consider possible interventions suggested by them to solve their issues according to their life context.

One priority area for intervention identified by the group, which was affecting all aspects of a professional’s life, was the balance between motherhood, marriage, and work, which was creating an overload for her colleague. A study comparing the time spent caring for babies, the division of household tasks, and the social support network of non-working mothers and mothers who work outside the home showed that working mothers set aside more time for childcare on weekends, while non-working mothers dedicated more time Monday through Friday. Concerning caregiving, the mother stood out as the primary caregiver, followed by the father, followed by the grandmother^(27)^.

Finally, although the professional rated some areas of her life at WL positively, the group’s feedback revealed otherwise, given their close acquaintance with the colleague. This finding highlights the importance of the therapeutic relationship between the team and patients in identifying care needs, which is a recommendation for PCCM operationalization^(12)^.

### Study limitations

A limitation of this study is the lack of physician participation in PCCM training workshops, which hinders the consolidation and standardization of practices based on person-centered care among all members of multidisciplinary teams. This scenario requires future research with this professional category to elucidate their approach to community mental healthcare service users, based on the biomedical model or person-centered psychosocial care.

### Contributions to nursing, health, or public policy

The research offers contributions to all professional categories working at CAPS, as it demonstrated in a practical way that WL can be used as a guiding principle for understanding the mental health-illness process and developing the PTS of individuals receiving care to manage their problems. The technique allows mapping priority areas of life for more immediate interventions, which promotes person-centered care.

## FINAL CONSIDERATIONS

The study demonstrated that WL is a tool that can be combined with CHE processes by encouraging professionals to reflect on the importance of practicing self-care so they can also care for others. Furthermore, it enabled them to identify what they were already doing to improve what was not working so well in their lives. Furthermore, the research demonstrates the relevance of using WL during nurses’ training, enabling them to equip themselves to apply this tool throughout their professional experience to care for individuals, families, and communities.

## Data Availability

The research data are available within the article.
