# Supplementary material for: Wheel of Life: self-care and continuing education tool for mental healthcare professionals
Source: Rev Bras Enferm. 2025 Nov 20;78(Suppl 3):e20250149. doi: 10.1590/0034-7167-2025-0149 (PMC12622975; doi:10.1590/0034-7167-2025-0149)
Supplement: Translation

# Translation: Roda da Vida: instrumento de autocuidado e educação continuada para profissionais de saúde mental

**DOI:** 10.1590/0034-7167-2025-0149pt

**Authors:** Johnatan Martins Sousa, Joyce Soares Silva Landim, Marciana Gonçalves Farinha, Roselma Lucchese, Camila Cardoso Caixeta, Thatianny Tanferri de Brito Paranaguá, Fernanda Costa Nunes, Ana Lúcia Queiroz Bezerra

**Affiliations:** I Universidade Federal de Goiás. Goiânia, Goiás, Brasil; II Universidade Federal de Uberlândia. Uberlândia, Minas Gerais, Brasil; III Universidade Federal de Catalão. Catalão, Goiás, Brasil; IV Universidade de Brasília. Brasília, Distrito Federal, Brasil

**Keywords:** Autocuidado, Vida, Assistência Centrada no Paciente, Educação Continuada, Serviços Comunitários de Saúde Mental.

## Abstract

**Objetivos:**

analisar o uso da Roda da Vida como instrumento de autocuidado e educação permanente em saúde para profissionais de saúde mental no cenário da atenção psicossocial.

**Métodos:**

pesquisa-intervenção qualitativa norteada pelo Ciclo de Aprendizagem Vivencial. Foram realizadas quatro oficinas com 30 profissionais de dois Centros de Atenção Psicossocial da região central do Brasil. Para a construção dos dados, foram utilizados questionário de perfil profissiográfico, Roda da Vida e anotações em diário de campo. Os dados foram submetidos à análise de conteúdo temática.

**Resultados:**

emergiram duas categorias: Repercussões da Roda da Vida para o autocuidado; e Roda da Vida como instrumento para o manejo dos problemas.

**Considerações Finais:**

o estudo evidenciou que a Roda da Vida é uma ferramenta que pode ser aliada a processos de educação permanente em saúde, além de promover reflexões nos profissionais sobre a importância de praticar o autocuidado para cuidar do outro.

## INTRODUÇÃO

A busca constante pelo conhecimento é algo inato a todo ser humano inserido na sociedade que procura respostas para diversas questões. Entretanto, a acomodação alicerçada no senso comum e em preconceitos arraigados no meio social pode prejudicar a construção de novos saberes, pois as pessoas passam a acreditar em “verdades” inquestionáveis^(1)^.

Para transformar esse cenário de estagnação no contexto do trabalho, a Educação Permanente em Saúde (EPS) é um recurso que gera mudanças na formação de diversos profissionais, aliando instituições formadoras, gestão dos serviços de saúde e equipes para aperfeiçoar os processos de trabalho, pois evidências científicas apontam a importância de ações de EPS em serviços comunitários de saúde mental para o aperfeiçoamento das práticas e implementação do cuidado centrado na pessoa^(2)^.

Nessa direção, pesquisadores sinalizam a importância de transformações e inovações nas metodologias de ensino para uma prática crítica, reflexiva e que respeite a singularidade do outro, incluindo a formação em saúde mental^(3)^. Uma estratégia que pode ser utilizada em processos de EPS é a Roda da Vida (RV), como demonstra estudo que utilizou essa ferramenta para promover o desenvolvimento da liderança em enfermeiros da Atenção Primária à Saúde (APS)^(4)^.

A RV é uma ferramenta gráfica em formato de pizza com escala de 1 a 10, em que a pessoa deverá avaliar de forma qualitativa e atribuir uma pontuação para cada área da sua vida de forma biopsicossocial para subsidiar intervenções para os aspectos analisados^(5)^.

Além de ser um instrumento mediador em processos de EPS, a RV pode ser considerada uma ferramenta de autocuidado porque permite que a pessoa que a realiza consiga identificar as potencialidades e fragilidades de todas as áreas de sua vida no momento presente. Pesquisa descreveu as repercussões de uma intervenção com Reiki no bem-estar e na saúde autorreferida de profissionais de enfermagem que utilizaram a RV como instrumento de coleta de dados, demonstrando que, após a intervenção, houve melhora do aspecto emocional dos participantes^(6)^.

No cenário da atenção psicossocial, são primordiais ações de autocuidado para os profissionais para promoção da saúde física e mental das equipes. Estudo observacional transversal, exploratório e descritivo, que descreveu o estilo de vida e a saúde mental de 47 trabalhadores dos Centros de Atenção Psicossocial (CAPS) I e II da região norte do estado do Tocantins, Brasil, revelou que apesar de a maioria ter sido classificada com boa qualidade de vida, foram identificados transtornos mentais comuns no grupo. Também se identificou que a prevalência encontrada foi superior à de outros estudos com profissionais da área da saúde^(7)^.

Diante do exposto, pesquisas sobre a construção de conhecimento sobre estratégias, métodos e ações que envolvem o planejamento e implementação da EPS ainda são escassas^(8)^. Tendo em vista que não basta apenas promover processos formativos junto às equipes de saúde mental, é importante aliar esses momentos de construção de saberes com práticas de autocuidado, especialmente no contexto da saúde mental, pois evidências científicas apontam que os profissionais de saúde mental estão suscetíveis a adoecimento relacionado ao ambiente de trabalho^(9)^. Logo, almejou-se responder à seguinte questão norteadora: quais as repercussões do emprego da RV para o autocuidado e EPS de profissionais de CAPS?

## OBJETIVOS

Analisar o uso da RV como instrumento de autocuidado e EPS para profissionais de saúde mental no cenário da atenção psicossocial.

## MÉTODOS

### Aspectos éticos

A pesquisa faz parte de projeto âncora aprovado pelo Comitê de Ética em Pesquisa do Hospital das Clínicas da Universidade Federal de Goiás. Foram seguidas as orientações da Resolução nº 466 de 2012 do Conselho Nacional de Saúde e, para conservar o anonimato dos participantes, todos foram codificados pela letra P e ordem de fala nos encontros grupais (P1 a P30).

### Tipo de estudo

Trata-se de pesquisa-intervenção de abordagem qualitativa, que é indicada quando se almeja compreender os sentidos, experiências e vivências de diversos atores dentro das instituições, associando uma intervenção psicossociológica no decorrer do estudo para estimular uma transformação institucional^(10)^. O estudo foi norteado pelo Ciclo de Aprendizagem Vivencial (CAV)^(11)^ e pelo referencial teórico do Método Clínico Centrado na Pessoa (MCCP)^(12)^. A descrição do relatório do estudo seguiu as recomendações do guia *COnsolidated criteria for REporting Qualitative research* ^(13)^.

### Cenário do estudo

A pesquisa foi realizada em dois CAPS da região central do Brasil, sendo um Centro de Atenção Psicossocial álcool e outras drogas (CAPSad) do tipo III e um Centro de Atenção Psicossocial infantojuvenil (CAPSi).

### Fonte de dados

Participaram 30 profissionais, 15 de cada CAPS, selecionados por conveniência, segundo o critério de inclusão de estarem prestando atendimento aos usuários e seus familiares. Foram excluídos os colaboradores que estavam em afastamento oficial do serviço por motivo de férias ou licenças, ou que atuavam, exclusivamente, em atividades administrativas.

### Coleta e organização dos dados

Para a construção dos dados, foram implementados quatro encontros grupais, de outubro a dezembro de 2022, no formato de oficina que seguiram os passos do CAV: atividade (implementação de uma vivência); análise (diagnóstico da situação que foi vivenciada pelo *feedback* deixado pelo grupo); conceituação (exposição de teoria sobre o tema abordado); e conexão (estabelecimento de relações de tudo que foi vivenciado com a vida pessoal e profissional)^(11)^. Todos os encontros tiveram duração de três horas, ocorreram com intervalo de 15 dias e aconteceram na sala de reuniões do CAPSad III, para onde os profissionais do CAPSi se deslocaram.

Os dados deste estudo são oriundos do terceiro encontro do processo formativo, que teve como finalidade trabalhar, junto aos profissionais, o segundo e terceiro componentes do MCCP: “Entendendo a pessoa como um todo”; e “Elaborando um plano conjunto de manejo dos problemas”^(12)^.

No início do encontro, foi disponibilizado lanche para os integrantes do grupo, material de papelaria para que confeccionassem um crachá pessoal, e foi entregue o questionário de perfil profissiográfico para preenchimento e o Termo de Consentimento Livre e Esclarecido. Logo em seguida, como estratégia de acolhimento e aquecimento, foi sugerido ao grupo a técnica “Meu superpoder”, em que foi disparada a pergunta: pensando no seu papel de profissional de saúde mental, que superpoder gostaria de ter? Foi entregue uma filipeta para cada profissional para registrar seu superpoder e depois cada um explicou o porquê da escolha do poder.

Na etapa da atividade do CAV, foi proposta para os profissionais a técnica da RV^(14)^ para trabalhar o segundo componente do MCCP, com o intuito de analisar as dimensões da vida dos integrantes do grupo, incluindo espiritualidade, saúde e disposição, desenvolvimento intelectual, equilíbrio emocional, carreira e trabalho, finanças, contribuição social, amizades e família, afetividade e amor, vida social, *hobbies* e diversão, e realização e felicidade. Cada profissional recebeu uma folha com a RV, canetinhas e lápis de cor para pintar a sua roda no tempo de dez minutos.

Após a conclusão da RV, foi perguntado ao grupo se alguém gostaria de expor a sua produção para os demais, orientado pelos seguintes questionamentos: como foi vivenciar a atividade? Qual é a relação do que foi vivenciado com a sua vida pessoal e trabalho?

Posteriormente, para trabalhar o terceiro componente do MCCP, o grupo foi convidado a construir o Projeto Terapêutico Singular (PTS) de um dos participantes de forma coletiva, tendo como eixo norteador a RV, e uma profissional se voluntariou. Após a sistematização do PTS para a problematização do tema abordado, foram disparadas para o grupo as seguintes questões: como foi vivenciar a atividade? Quais as dificuldades e facilidades? Como vocês constroem coletivamente o cuidado com o usuário? Quais estratégias? Ferramentas? Os usuários e familiares são incluídos nesse processo? Quem elabora esse plano de cuidados?

Os encontros foram mediados por dois profissionais: um enfermeiro mestre, especialista em saúde mental, enfermagem psiquiátrica e dinâmica de grupo e gestão de equipes; e uma psicóloga, doutora em ciências da saúde e especialista em consultoria e gestão de grupos.

### Análise dos dados

Todo o processo foi registrado em formato de áudio e registros em diário de campo, e foi submetido à análise de conteúdo temática de acordo com as etapas preconizadas por Bardin^(15)^: pré-análise, marcada pela organização e seleção dos materiais que serão analisados, bem como pela leitura flutuante dos dados para subsidiar as hipóteses iniciais; exploração do material, que consiste na codificação dos dados por meio da identificação das unidades de registro e contexto, que são agrupadas por semelhança para a construção dos núcleos de sentido; e tratamento dos resultados: inferência e interpretação, que é a divulgação das informações analisadas por meio das categorias 1 (Repercussões da Roda da Vida para o autocuidado) e 2 (Roda da Vida como instrumento para o manejo dos problemas).

## RESULTADOS

### Caracterização sociodemográfica

Vinte e oito dos 30 profissionais que participaram do estudo eram do sexo feminino (93%). A faixa etária mais numerosa foi de 30 a 49 anos de idade (19 participantes; 63%), e as formações profissionais foram diversas (psicólogo (11), técnico de enfermagem (8), enfermeiro (3), farmacêutico (2), fisioterapeuta (2), assistente social (1), pedagogo (1), musicoterapeuta (1), profissional de educação física (1)).

### Análise de conteúdo

Como fruto do processo de análise de conteúdo, foram construídas duas categorias (1. Repercussões da Roda da Vida para o autocuidado; 2. Roda da Vida como instrumento para o manejo dos problemas), que demonstram os desdobramentos do emprego da RV com as equipes de saúde mental.

### Categoria 1. Repercussões da Roda da Vida para o autocuidado

Os participantes verbalizaram que, por meio da RV, conseguiram perceber a importância de se reconhecerem como ser humano e que também têm fragilidades como as pessoas que atendem:

*Quando eu trabalhava na UBS, eu estava com febre e eu fui fazer triagem, e uma paciente me olhou e falou: “Uai, doutora também adoece?”, e aí eu falei pra ela: “Primeiro, que eu não sou doutora, segundo que eu sou de carne e osso igual a você”.* (P2)

*E gente, é ótimo você se tornar humano, para se encontrar com outro humano, que é esse lance de cuidado centrado na pessoa, e é muito legal.* (P6)

A RV permitiu ao grupo perceber que, mesmo estando no papel de cuidadores, os profissionais também são passíveis de adoecimento e que a aceitação de tais fragilidades pessoais humaniza o atendimento:

*Antes, a minha insegurança que a gente tem de profissional recente, era isso, do tipo “Nossa, eu não posso adoecer. Eu tenho que ser forte e tal”, mas, com a experiência de vida, a gente vê que não; quanto mais humano e doente você é, mais você faz vínculo, você cria vínculo com seu paciente* [...]. (P6)

Entre os profissionais, um participante expôs que usa o recurso da RV em seus atendimentos e que a sua efetividade é potencializada quando ele socializa experiências próprias com os adolescentes, para que eles se sintam confiantes para revelar as suas questões:

*E é uma técnica boa, tanto para o individual quanto em grupo. Eu trabalho com adolescentes, que a gente tenta extrair as informações dele, mas se a gente só tentar extrair informações sem dar nada em troca, eles se fecham. E aí, o que que eu faço? Eu converso com eles, conto minha história de vida, o que aconteceu comigo e, depois disso, eles começam a devolver.* (P12)

A RV se mostrou pertinente pois permitiu o diagnóstico situacional do momento atual da vida dos profissionais por meio da identificação das áreas mais satisfatórias e as que precisavam de maior atenção pelos participantes:

[...] *capenga, porque, realmente, tem umas áreas que eu coloquei aqui* [...] *o equilíbrio emocional, eu coloquei no máximo, mas aí, quando eu olhei para minha saúde e disposição, eu percebi que a saúde e a disposição fazem parte desse equilíbrio emocional. Aí eu vi uma discrepância, falei: “Opa”, me fez refletir que para eu estar aqui nesse dez de equilíbrio emocional. Eu tenho que voltar lá na minha saúde e cuidar mais dela, porque também isso vem aqui para o equilíbrio emocional* [...]. (P8)

A RV fez com que um participante conseguisse visualizar possíveis causas que estava prejudicando com que a sua roda girasse, como os medos do passado que atrapalhava a sua vida amorosa:

*E a questão da afetividade, que foi o que a Selma falou também, há quanto tempo eu não me abro para um relacionamento com medo de acontecer o que aconteceu antes? Então, essas coisas, né. Mas, assim, é legal olhar as coisas que podem mudar* [...]. (P2)

Emergiram nos depoimentos dos participantes que a RV os sensibilizou para questões como autoconhecimento, necessidade de olhar para si mesmo, levar em consideração a própria perspectiva, não o que a sociedade dita:

*Eu tentei focar mais em mim, assim, de não olhar tanto* [...] *porque, se eu fosse olhar pelo o que a própria sociedade cobra, eu acho que estaria bem abaixo.* (P11)

*Eu acho muito interessante essa experiência da gente fazer a Roda da Vida, porque, para mim, foi um momento que eu fiquei mais calada, porque foi uma coisa mais reflexiva. Porque eu tenho observado a minha vida, mas quando você olha no papel, você tá lá refletindo aquilo ali, você tá tendo noção do que você não sabe, para você realmente ver que áreas que tá pegando* [...]. (P7)

Um dos profissionais trouxe à tona a reflexão de que é preciso estar bem consigo mesmo para que possa cuidar dos usuários:

[...] *eu fiquei mais reflexiva por conta disso, dessas coisas que você vê que mexe com seu emocional. Você fala: “Nossa, aqui tá ruim”. Isso quando você falou, quando a gente coloca as nossas emoções, aquilo que você está vivendo, e você vê que melhora em você, reflete na pessoa que você tá atendendo* [...]. (P7)

A RV chamou a atenção para a necessidade de colocar em prática a empatia durante a assistência à saúde mental, pois todas as pessoas apresentam dificuldades durante a sua trajetória de vida:

*Foi muito bom, porque a gente lida com muito problema com o usuário internar e sair no mesmo dia* [...] *e, para nós profissionais, isso é frustrante. Ele vai voltar para a rua, ele vai voltar a dar trabalho para a família e para a sociedade. Então, foi bom o papo, porque a gente percebe que é ser humano, cada um tem seu momento, tem as variáveis, então foi muito proveitoso para mim e para as colegas, porque a gente conversou aqui entre a gente.* (P23)

Outra importante contribuição da RV foi o reconhecimento por um dos participantes do que já estava sendo feito para a melhoria do que não estava tão bom em sua vida:

*No meu caso, eu necessito de saúde e disposição. Já estou em movimento, já estou no processo com nutrólogo. Então, esse é o meu ponto, é a saúde. Já comecei uma intervenção. No meu caso mesmo, eu mexendo aqui, vai mexer os outros. Se eu mexer nisso, vai melhorar.* (P8)

### Categoria 2. Roda da Vida como instrumento para o manejo dos problemas

Após todos os integrantes do grupo terem realizado a sua RV, uma participante permitiu a utilização da sua produção para construção coletiva de um PTS para explicar de forma prática o terceiro componente do MCCP: “Elaborando um plano conjunto de manejo dos problemas”. A Figura 1 apresenta a RV da profissional.

**Figura 1:** Roda da Vida de uma profissional integrante do grupo, Aparecida de Goiânia, Goiás, Brasil, 2022

*Fonte: Pavlo^(14)^.*

Por intermédio da RV, a profissional conseguiu identificar quais áreas de sua vida estavam mais críticas como finanças, carreira e trabalho, vida social, e *hobbies* e diversão:

*O financeiro não está legal, não tenho vida social, não tem roda de diversão, nem dinheiro, as finanças estão lascadas.* (P3)

*O social, a diversão estão mais lascados mesmo.* (P3)

*Eu trabalho, trabalho, trabalho. Carreira e trabalho está mais alto.* (P3)

Ao mostrar para o grupo que atribuiu uma nota muito alta para o campo do equilíbrio emocional, o *feedback* dos colegas evidenciou contradições entre o dito e o real:

*E como o seu equilíbrio está alto?* (P12)

*O meu marido falou que eu estou muito agressiva. Eu falei: “Rapaz, não fala que eu estou agressiva, não”.* (P3)

Outra área da vida que apresentou contradições entre a pontuação elevada e a forma como realmente se apresentam foi a questão da felicidade e realização, em que um colega de trabalho que atua com a profissional sinalizou incongruências:

*Eu nem tinha visto isso aqui, a vida dela desse jeito, e a realização e felicidade dela está plena.* (P6)

A RV favoreceu que a profissional pudesse visualizar quais áreas de sua vida precisam de maiores investimentos, como lazer e diversão, e também possibilitou a reflexão de possíveis soluções como a conciliação entre a carreira e a maternidade para a melhora das finanças:

*Tem que melhorar, mas, para melhorar, tem que melhorar outras coisas primeiro. Finanças, carreira e trabalho deslanchar.* (P3)

*Agora nesse momento, eu preciso conciliar com a maternidade. Eu tenho uma bebê de 8 meses e preciso trabalhar, mas tenho que conciliar com meu esposo, com o tempo dele, porque ela fica comigo e com ele* [...] *a gente fica revezando.* (P3)

Realizar concessões em seu projeto de vida em detrimento dos desejos de outras pessoas, como o marido, também foi sinalizado pela profissional no momento de problematização de sua RV:

[...] *mas agora ele* [meu marido] *não quer mais trabalhar por conta própria; ele quer CLT. E aí, para ele trabalhar CLT, eu vou ter que abrir mão de um dos trabalhos. Eu tenho dois trabalhos.* (P3)

Outra situação paradoxal evidenciada na RV foram pontuações expressivas em relação à afetividade e amor. Entretanto, a profissional vocalizou a ausência de momentos particulares com o marido e que a maioria do seu tempo está depositado no trabalho e nos cuidados com a filha:

*Plena, aqui tá plena. Afetividade, amor, amizade, família.* (P3)

*Na afetividade, no amor, eu acho que eu olhei mais para a questão da minha família, da minha filha, dos amigos e meio que um respondeu o outro, né.* (P3)

Sobre o campo das amizades, a profissional expressou que, para não enfraquecer o vínculo, convida os colegas de trabalho para visitar a sua casa ou realizar chamadas de vídeo:

*Convivo com amigos no trabalho, chamada de vídeo, quando alguém vai lá em casa.* (P3)

Os facilitadores perguntaram para a profissional se a RV fosse a roda do tempo, qual seria a área que teria maior pontuação, obtendo como resposta o trabalho. Posteriormente, foi indagado se fosse a roda, o que seria mais importante, e ela respondeu “família”. Diante dessas respostas, foi perguntado se uma roda tivesse que sobrepor a outra se seria possível, a profissional balançou a cabeça com sinal de negativo:

*Tem que achar um caminho para conciliar.* (P3)

Posteriormente à exposição da RV pela profissional, os facilitadores disponibilizaram duas folhas com cópias da estrutura do PTS e que também foi projetada na parede da sala por meio de Datashow para a formulação de estratégias de cuidado por todo o grupo:

*Como amiga íntima, quando a gente propõe alguma coisa, aí ela fala assim: “Não, vem aqui para casa”. É mais fácil receber lá do que sair. A gente queria que ela fosse para o parque com a neném, mas a gente vê aqui, eu acho que na verdade, não é nem por falta de condições, mas para ela estar assim triste, cansada.* (P2)

*Aí eu anotei como possíveis situações-problema o cansaço físico, pouco sono, privação de sono, sono pouco reparador, dificuldade de expressar, de pedir ajuda, de buscar ajuda, insatisfação, tristeza, esgotamento, dificuldade, isolamento, falta de convivência social, dificuldade financeira, diminuída ou a ausência de rede de apoio e pouco tempo para si mesma.* (P6)

Para aliviar a sobrecarga, o grupo sugeriu possibilidades de ações para a minimização dos prejuízos, como viabilizar a matrícula da filha em um Centro Municipal de Educação Infantil (CMEI):

*E aí, quando a gente coloca isso, eu vou falar nesse descanso, e aí, como é possível, na configuração atual, ter o descanso se a gente pensa na ausência de rede de apoio? Que não tem família? Então, como pode ter rede de apoio? Então, tenho que adicionar CMEI, que vai ser o tempo para cuidar desse bebê e que vai dar tempo para ela e do marido, de forma a ela se sentir menos culpada e que ela vai poder se organizar, a partir daí, em detrimento de outras coisas.* (P6)

Outra possibilidade levantada foi o revezamento entre amigos para o cuidado da bebê, porém a profissional salientou que, por causa do comportamento ciumento, o pai não autoriza:

*E aí, tem toda essa questão de o pai de ver o que é esses ciúmes, essa insegurança em deixar a filha com pessoas desconhecidas. Poderia ver quem são as pessoas do ciclo de amigos que ele confiaria, para poder compartilhar e tal. E aí, tem outra demanda, que é pensar nas estratégias para além do CMEI.* (P6)

Foram sugeridos cuidados em saúde mental para a profissional, pois a mesma apresenta resistências em pedir ajuda, o que pode desencadear sofrimento emocional:

*Outra coisa também seria urgentemente encaminhar para o Núcleo de Saúde Mental. Ela precisa de uma terapia, porque ela tem esse movimento de se fechar muito, de não dar conta de pedir ajuda, de querer dar conta de tudo sozinha. Então, emocional dela que ficaria aliviado se ela permitisse não dar conta, não se cobrar tanto ou entender um pouco mais esse movimento seja mais compassiva consigo mesma, abrir o espaço.* (P6)

Mesmo com todos os esforços para construir um PTS assertivo para a colega, emergiu no grupo a importância de levar em consideração o desejo da profissional de aderir ou não ao que foi formulado, o que se alinha ao cuidado centrado na pessoa:

*A gente tá falando do tempo, porque a neném vai estar no CMEI. E aí, a gente vai traçar os níveis de prioridade. Se você quiser, você pode pensar nisso também, até porque a gente pensa assim: “Tem coisas que são ideais, estão palpáveis, estão à disposição, agora você que vai ver se você quer ou não isso daqui que são possibilidades”.* (P6)

## DISCUSSÃO

A categoria “Repercussões da Roda da Vida para o autocuidado” evidenciou que a utilização dessa ferramenta com os profissionais de saúde foi para que se enxergassem como seres humanos, igualmente aos usuários dos CAPS. O autoconceito que a pessoa tem de si mesma, por meio das suas percepções, crenças, valores, competências, entre outros, é o que influencia o modo como cada indivíduo se comporta no meio em que está inserido^(16)^. Logo, ao se reconhecer como um ser humano diante das pessoas que atende, o cuidado em saúde mental se torna mais humanizado.

A RV também fez com que os profissionais de saúde mental reconhecessem que também estão sujeitos ao adoecimento, assim como as pessoas que atendem. Revisão integrativa da literatura que descreveu o processo de saúde-doença nos profissionais de saúde mental revelou que as condições de trabalho podem interferir na qualidade de vida desses profissionais e que não foram encontrados estudos recentes que abordaram essa temática na literatura científica^(17)^.

Foi externado que a RV também é utilizada por um dos profissionais com adolescentes e, quando ele expõe as suas questões, o usuário se sente mais confortável em falar sobre a sua vida. Em um processo terapêutico, quando a pessoa é aceita incondicionalmente da forma que é, é tratada de forma empática e se sente em um ambiente seguro; ela caminha para o desenvolvimento da autonomia pessoal e passa a se responsabilizar por si mesma, tornando-se livre^(18)^. Logo, ao utilizar a RV, o profissional também olha para si mesmo e reflete sobre a sua vida, o que fortalece o vínculo com o usuário.

A RV permitiu que os participantes identificassem as áreas de sua vida que estavam melhores em relação a outras que precisavam de maiores investimentos, bem como a visualização de possíveis causas do que não estava tão bom naquele momento atual. O movimento de voltar-se para o momento presente é uma atividade pouco praticada. Uma possibilidade que favorece o fixar-se no presente e que pode ser utilizada pelos profissionais é a RV, como demonstra estudo realizado com estudantes de medicina que utilizaram esse recurso para identificar os pontos fortes e vulneráveis do atual momento de vida de uma paciente no contexto da APS^(19)^.

A RV despertou os profissionais para reflexões sobre autoconhecimento, voltar-se para si, olhar para sua própria perspectiva, não para o que a sociedade prega. No contexto da assistência à saúde, o autoconhecimento é um dos atributos desejáveis para implementação do cuidado centrado na pessoa, pois o profissional que atende uma pessoa acometida por alguma doença só é capaz de compreendê-la se conhecer primeiramente a si mesmo^(12)^.

A intervenção realizada proporcionou ao grupo perceber que, para cuidar do outro, primeiramente é necessário cuidar de si. Os profissionais de saúde também estão sujeitos a sofrimento psíquico e precisam se submeter a cuidados^(20)^. Logo, o autocuidado é essencial tanto para a manutenção da saúde física quanto mental das equipes que lidam de forma rotineira com o sofrimento de outras pessoas.

Outra repercussão da RV no grupo foi a sensibilização para exercer a empatia durante sua prática laboral nos CAPS. Um aspecto primordial para a prática do cuidado centrado na pessoa é o emprego da empatia por quem está prestando a assistência para indivíduos, famílias e comunidades^(21)^. Assim, faz-se necessário que as equipes multiprofissionais que atuam nos CAPS invistam nessas tecnologias de cuidado relacionais para uma melhor assistência à saúde mental.

Além de realizar o diagnóstico situacional da vida dos profissionais, a RV também possibilitou que eles visualizassem o que já estava sendo feito por eles para mudar essa realidade. A RV é um instrumento de avaliação que permite analisar as principais áreas da vida que são fatiadas e representadas visualmente em forma gráfica, que representa o olhar da pessoa sobre a forma como está a sua vida naquele momento e como gostaria que ela estivesse^(22)^.

A categoria “Roda da Vida como instrumento para o manejo dos problemas” demonstrou, por meio da explanação da RV de um dos profissionais, que esse recurso pode ser utilizado para esta finalidade nos CAPS. A técnica possibilitou com que a pessoa identificasse as áreas críticas de sua vida que, consequentemente, afetam a sua saúde mental.

Para que uma pessoa tenha de fato saúde mental, não basta apenas a ausência de transtornos mentais ou sofrimento psíquico. A saúde mental envolve o equilíbrio entre determinantes internos e externos ao ser humano, o que engloba a família, trabalho, vida em sociedade, relações amorosas e a forma de lidar com as frustrações. Sendo assim, o equilíbrio entre todos esses aspectos confere saúde mental para a pessoa^(19)^.

A RV fez com que a profissional parasse para pensar sobre a sua própria vida, o que, muitas vezes, pelas inúmeras demandas e atribuições do cotidiano, esses momentos não são reservados para o autocuidado. Na contemporaneidade, devido à correria do dia a dia e pela busca desenfreada das pessoas por algo que está fora de si, o sujeito acaba entrando em um estado de sofrimento, pois acaba esquecendo de si mesmo e de suas emoções, o que gera anulação de si mesmo e desencadeia um comportamento robotizado^(23)^. Logo, são importantes momentos de reflexão sobre a própria vida para resgatar o que é realmente importante para a pessoa e sair do automatismo.

A RV possibilitou que a profissional contasse qual a área de sua vida estava com maior pontuação relacionada ao trabalho em detrimento das outras. O estresse ocasionado pelo trabalho é um fenômeno que gera desequilíbrio na vida das pessoas, pois cobrança excessiva, nervosismo, esgotamento extremo e irritabilidade podem ocasionar adoecimento com alterações de humor, diminuição da produtividade, prejuízos nos relacionamentos interpessoais e na qualidade de vida, o que inviabiliza a manifestação plena da felicidade^(23)^.

Esse achado demonstra uma anulação da pessoa que acaba gerando sofrimento. Nesse sentido, ao praticarem o cuidado centrado na pessoa, é importante que os profissionais valorizem e respeitem as preferências de quem estão atendendo, não a conveniência da equipe^(21)^. Ao ignorarem a real vontade dos usuários, os trabalhadores estarão reforçando esse movimento de passividade da pessoa diante de sua própria vida.

A RV da profissional também evidenciou a dificuldade de conciliação da maternidade com as demais áreas de sua vida, como o trabalho e a vida pessoal. Desde os primórdios, as mulheres sempre assumiram o papel de cuidadoras e, ao serem colocadas nesse lugar, muitas vezes não questionam, o que ficou arraigado na sociedade como um valor moral relacionado ao universo feminino. Mesmo com alguns avanços nos últimos tempos, os cuidados com os filhos e atividades domésticas ainda, em sua maioria, ficam sob responsabilidade das mulheres^(24)^. Quando as mulheres trabalham fora de casa, isso gera uma dupla jornada que consome o seu tempo para se dedicar às outras áreas da vida, como o relacionamento amoroso.

Sobre o campo das amizades, a profissional verbalizou que, para não prejudicar o vínculo com os amigos, convida-os para frequentar a sua casa. As relações de amizade são importantes para o desenvolvimento psicoemocional das pessoas e podem inclusive influenciar o desenvolvimento de transtornos mentais. Revisão integrativa que analisou a produção científica sobre relações de amizade em pessoas com transtornos alimentares revelou que o vínculo de boa qualidade reduziu a frequência dos sintomas, enquanto que amizades que verbalizaram comentários negativos em relação ao corpo e alimentação da pessoa favoreceram o estabelecimento dos transtornos^(25)^. Logo, é importante considerar que, como as demais áreas da vida, o campo das amizades também precisa de investimentos.

Após a apresentação da RV, o grupo construiu o PTS da colega de trabalho, pensando em intervenções para minimizar ou solucionar os problemas que emergiram. A RV é uma ferramenta que pode ser mediadora da construção do PTS, pois faz com que a própria pessoa que busca ajuda consiga refletir sobre o que é melhor para si, de acordo com a sua própria perspectiva^(19)^. Logo, esse movimento de voltar para si mesmo contribui para concretização do cuidado centrado na pessoa, não nos desejos e vontades apenas do profissional de saúde.

Nos CAPS, uma estratégia potente de formular planos de ação para atender às necessidades dos usuários é o PTS. O PTS, quando construído coletivamente, potencializa os vínculos estabelecidos entre a equipe, usuários e seus familiares, pois o envolvimento do usuário no processo é permitido para o desenvolvimento de sua autonomia durante a assistência à saúde^(26)^. Logo, é importante dar voz aos usuários, não apenas para identificar os seus problemas, mas também para considerar possíveis intervenções sugeridas por eles para solução de suas questões de acordo com o seu contexto de vida.

Uma área prioritária para intervenção que o grupo elencou, que estava afetando todas as dimensões da vida da profissional, foi o equilíbrio entre a maternidade, casamento e trabalho, que estava gerando sobrecarga na colega. Pesquisa que comparou o tempo de cuidado com o bebê, divisão de tarefas domésticas e rede de apoio social de mães que não trabalham e de mães que trabalham fora de casa evidenciou que mães que trabalham dedicam mais tempo aos cuidados com os filhos nos fins de semana e mães que não trabalham dedicam mais tempo a isso de segunda a sexta-feira. Já em relação aos cuidados, a mãe se destacou como a principal cuidadora, seguida pelo pai e pela avó^(27)^.

Por fim, apesar de a profissional ter pontuado positivamente algumas áreas de sua vida na RV, o *feedback* do grupo revelou o contrário pelo fato de conhecerem de perto a colega. Esse achado chama a atenção para a importância da relação terapêutica entre a equipe e os usuários para a identificação das necessidades de cuidado, o que é uma recomendação para a operacionalização do MCCP^(12)^.

### Limitações do estudo

Como limitação do estudo, destaca-se a não participação dos médicos nas oficinas de formação para o MCCP, o que prejudica a consolidação e uniformização de práticas baseadas no cuidado centrado na pessoa por parte de todos que compõem as equipes multiprofissionais. Tal cenário requer pesquisas futuras com essa categoria profissional, para elucidar como é a sua abordagem aos usuários dos serviços comunitários de saúde mental, baseadas no modelo biomédico ou na atenção psicossocial centrado na pessoa.

### Contribuições para as áreas da enfermagem, saúde ou políticas públicas

A pesquisa traz contribuições para todas as categorias profissionais que atuam nos CAPS, pois demonstrou de forma prática que a RV pode ser utilizada como eixo norteador para a compreensão do processo de saúde-doença mental e construção do PTS das pessoas que são atendidas para o manejo dos problemas. A técnica permite mapear as áreas da vida prioritárias para intervenções mais imediatas, o que favorece o cuidado centrado na pessoa.

## CONSIDERAÇÕES FINAIS

O estudo evidenciou que a RV é uma ferramenta que pode ser aliada a processos de EPS ao promover reflexões nos profissionais sobre a importância de praticar o autocuidado para cuidar de si para que possam cuidar do outro também. Além disso, possibilitou identificar o que já estavam fazendo para melhorar aquilo que não estava tão bom em suas vidas. Ademais, a pesquisa demonstra a relevância da utilização da RV durante o processo de formação dos enfermeiros para que eles possam se instrumentalizar para aplicar essa ferramenta durante a sua vivência profissional para a assistência do indivíduo, família e comunidade.

## Data Availability

Os dados da pesquisa estão disponíveis no corpo do artigo.
